# 3D CoMoSe_4_ Nanosheet Arrays Converted Directly from Hydrothermally Processed CoMoO_4_ Nanosheet Arrays by Plasma-Assisted Selenization Process Toward Excellent Anode Material in Sodium-Ion Battery

**DOI:** 10.1186/s11671-019-3035-6

**Published:** 2019-06-25

**Authors:** Shan Zhang, Yuanfei Ai, Shu-Chi Wu, Hsiang-Ju Liao, Teng-Yu Su, Jyun-Hong Chen, Chuan-Hsun Wang, Ling Lee, Yu-Ze Chen, Binbin Xu, Shin-Yi Tang, Ding Chou Wu, Shao-Shin Lee, Jun Yin, Jing Li, Junyong Kang, Yu-Lun Chueh

**Affiliations:** 1 Collaborative Innovation Center for Optoelectronic Semiconductors and Efficient Devices, Pen-Tung Sah Institute of Micro-Nano Science and Technology/Department of Physics, Xiamen University, Xiamen, 361005 Fujian China; 2 Department of Materials Science and Engineering, National Tsing Hua University, Hsinchu, 30013 Taiwan, Republic of China; 3 Frontier Research Center on Fundamental and Applied Sciences of Matters, National Tsing Hua University, Hsinchu, 30013 Taiwan, Republic of China; 4 College of Chemistry and Chemical Engineering, Xiamen University, Xiamen, 361005 Fujian China; 5 Department of Physics, National Sun Yat-Sen University, Kaohsiung, 80424 Taiwan, Republic of China

**Keywords:** CoMoSe_4_ nanosheet arrays, CoMoO_4_ nanosheet arrays, Plasma-assisted selenization, Sodium-ion battery

## Abstract

In this work, three-dimensional (3D) CoMoSe_4_ nanosheet arrays on network fibers of a carbon cloth denoted as CoMoSe_4_@C converted directly from CoMoO_4_ nanosheet arrays prepared by a hydrothermal process followed by the plasma-assisted selenization at a low temperature of 450 °C as an anode for sodium-ion battery (SIB) were demonstrated for the first time. With the plasma-assisted treatment on the selenization process, oxygen (O) atoms can be replaced by selenium (Se) atoms without the degradation on morphology at a low selenization temperature of 450 °C. Owing to the high specific surface area from the well-defined 3D structure, high electron conductivity, and bi-metal electrochemical activity, the superior performance with a large sodium-ion storage of 475 mA h g^−1^ under 0.5–3 V potential range at 0.1 A g^−1^ was accomplished by using this CoMoSe_4_@C as the electrode. Additionally, the capacity retention was well maintained over 80 % from the second cycle, exhibiting a satisfied capacity of 301 mA h g^−1^ even after 50 cycles. The work delivered a new approach to prepare a binary transition metallic selenide and definitely enriches the possibilities for promising anode materials in SIBs with high performances.

**Electronic supplementary material:**

The online version of this article (10.1186/s11671-019-3035-6) contains supplementary material, which is available to authorized users.

## Background

Rechargeable sodium-ion batteries (SIBs), benefiting from advantages of low cost and relatively high safety, have been considered as a promising alternative battery system to commercial lithium-ion batteries (LIBs) and received tremendous attention during the last decades [1–5]. Nevertheless, the larger ionic radius and higher molar mass of sodium ions compared with that of lithium ions lead to a sluggish electrochemical reaction for the sodium-ion diffusion, which consequently results in the unsatisfied electrochemical performances with less options on suitable electrode materials than those in LIBs [6–8]. Therefore, it is quite important to explore or design appropriate anode materials for SIBs.

Metal sulfides/selenides (MXs) have been demonstrated as very popular electrode material in SIBs because of their unique crystal structures and varieties in material properties [9–15]. Nonetheless, the large volume change in MXs during ionic extraction and insertion processes, generally resulting in structural degradation and instability of the solid electrolyte interphase, is still a serious issue. Therefore, further strategies are still needed to accommodate or buffer the material structures for practical applications [16, 17]. Recently, bimetallic sulfides/selenides, e.g., NiCo_2_S_4_, Co_2_Mo_3_Se, and CoMoS [18–20], have been investigated as a promising class of electrode materials for promising energy storage and conversion devices because of their higher electrochemical activities and capacities than mono-metal sulfides/selenides, e.g., MoS_2_, CoSe_2_, NiSe_2_, and FeSe_2_ [21–27]. However, in the field of SIBs, there have been few reports on the application of bimetallic selenides because of the challenge in material synthesis. Up to date, some synthetic methods and applications of bimetallic selenides in SIBs have been carried out [28–30]. Among them, Co and Mo, as transition metal elements with abundant resources and high redox chemical valences [31–35], are promising components as anode materials. Additionally, carbon cloth with highly textured surface and good electrical conductivity is a good substrate for electrode materials, which can enable fast electron transport and produce large electrode–electrolyte contact areas [37, 38].

In this regard, we demonstrated 3D-networked CoMoSe_4_ nanosheet arrays on network fibers of the carbon cloth (CoMoSe_4_@C) by direct chemical conversion through the plasma-assisted selenization of CoMoO_4_ nanosheet arrays prepared by the hydrothermal process on network fibers of the carbon cloth (CoMoO_4_@C) as the anode in SIBs for the first time. Interestingly, with the assistance of plasma-assisted process on selenization process, the conversion of O atoms by Se can be achieved at a low temperature of 450 °C without any morphology change. The CoMoSe_4_@C shows better sodium storage performance than that of the unselenized CoMoO_4_@C. With synergetic effects from both transition metal species, a highly reversible capacity of 475 mA h g^−1^ at 0.1 A g^−1^ and a high capacity retention of over 80% even after 50 cycles at 0.5 A g^−1^ were accomplished using the CoMoSe_4_@C composite as the electrode in SIBs. Furthermore, this composite electrode can deliver excellent rate capabilities with the discharge capacities changing from 475 to 230 mA h g^−1^ as current densities were stepwisely added ranging from 0.1 to 5 A g^−1^, exhibiting a good sodium storage property. This work developed a new pathway of synthesizing bimetallic selenides, which may be adopted in other related materials for the sodium energy storage or other applications [39–43].

## Experimental Section

### Synthesis of CoMoO_4_ Nanosheet Arrays by the Hydrothermal Process

Firstly, 0.4234 g Na_2_MoO_4_·2H_2_O (purity ≥ 99%, Sigma-Aldrich), 0.5093 g Co(NO_3_)_2_·6H_2_O (purity ≥ 98%, Alfa Aesar), 0.074 g NH_4_F (purity ≥ 98%, Alfa Aesar), and 0.49 g CO(NH_2_)_2_ (purity ≥ 99.5%, Echo Chemical Co., Ltd.) were added to 35 mL of distilled (DI) water under constant intense stirring. Then, the mixture was transferred to the Teflon-lined stainless autoclave, containing a piece of carbon cloth (CC) (CeTech Co., Ltd., Taiwan), followed by heating at 180 °C for 12 h in an oven. After the hydrothermal growth, the as-synthesized sample was taken out and carefully cleaned, followed by the vacuum-drying at 60 °C for 12 h. Finally, the as-synthesized sample was annealed in pure argon at 300 °C for 2 h to obtain the CC coated with CoMoO_4_ nanosheet arrays.

### Direct Conversion of CoMoSe_4_ Nanosheet Arrays by Plasma-Assisted Selenization Process

The plasma-assisted selenization system (Syskey Technology Ltd.) was used to selenize the as-produced CoMoO_4_ nanosheet arrays. The selenium heater at the top of the machine is separated from the lower sample holder to independently control the temperature of the Se source and substrate, respectively. In the synthesis process, the selenium particles were firstly placed on the selenium (Se) heater and were heated to 300 °C to generate Se vapors. At the same time, the vaporized Se gas was carried out to the substrate by a vertical flow of a mixed carrier gas containing N_2_/H_2_ gas (N_2_:H_2_ = 40:80) at the steady flow rate to maintain the amount of Se in the vapor. Subsequently, the substrate previously placed on the sample holder was heated to the reaction temperature of 450 °C. Once the substrate temperature was stable, the plasma was initiated at 250 W to ionize Se vapors into Se radials to promote the chemical reaction.

### Characterization

Morphologies of as-produced materials were characterized by scanning electron microscopy (SEM) (Hitachi UHR FE-SEM SU8010). Further observations of the difference in structures before and after the plasma-assisted selenization were examined using a high-resolution transmission electron microscope (HRTEM) (JEOL, JEM-F200 CFEGTEM, 200 kV). The elemental analyses were conducted by electron energy loss spectroscopy (EELS) via HRTEM (JEOL, JEM-F200). The formation of CoMoSe_4_@C was examined by Raman spectroscopy (HORIBA, LabRAM, HR800) with the green laser (532 nm) excitation. The crystal structures of CoMoO_4_ and CoMoSe_4_ were then characterized by X-ray diffraction (XRD) (Ultima IV, Rigaku). The chemical bonding and the depth profile of materials were established by X-ray photoelectron spectroscopy (XPS, ULVAC-PHI 1600) facility. Electrochemical testing of the prepared CoMoSe_4_@C was carried out using a CR2032 coin cell, consisting of a CoMoSe_4_@C electrode and a sodium metal cathode separated by glass fibers. CoMoSe_4_@C was directly used as an anode electrode, and its corresponding weight was calculated by subtracting the weight of the carbon cloth from the CoMoSe_4_@C composite. The electrolyte is 1 M sodium trifluoromethanesulfonate (NaCF_3_SO_3_) dissolved in diethyleneglycol dimethylether (DEGDME). To investigate the electrochemical performance of the assembled electrodes, cyclic voltammetry (CV) was performed in 0.5–3 V potential ranges at 0.1 mV s^−1^ on a Bio-Logic VSP potentiostat, and the electrochemical impedance was conducted using electrochemical impedance spectroscopy (EIS) over the frequency ranges of 0.01 Hz–100 kHz. Charging/discharging measurements were conducted under 0.5–3 V on Land Battery Measurement System at room temperature.

## Results and Discussion

**Scheme 1:**
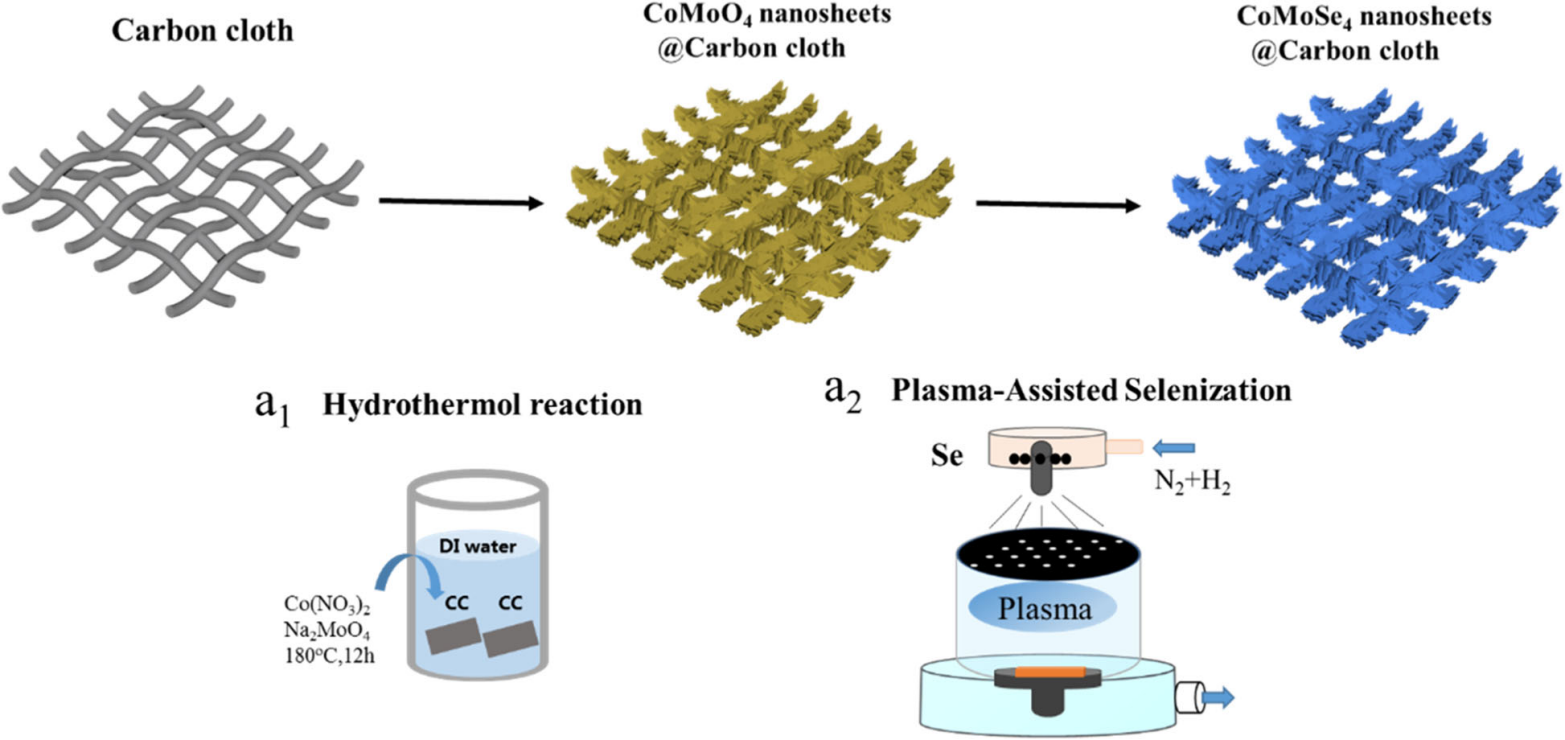
Schematic diagram of the fabrication processes of 3D CoMoSe_4_@C through the hydrothermal reaction (a_1_) and followed by the plasma-assisted selenization process (a_2_)

**Fig. 1:**
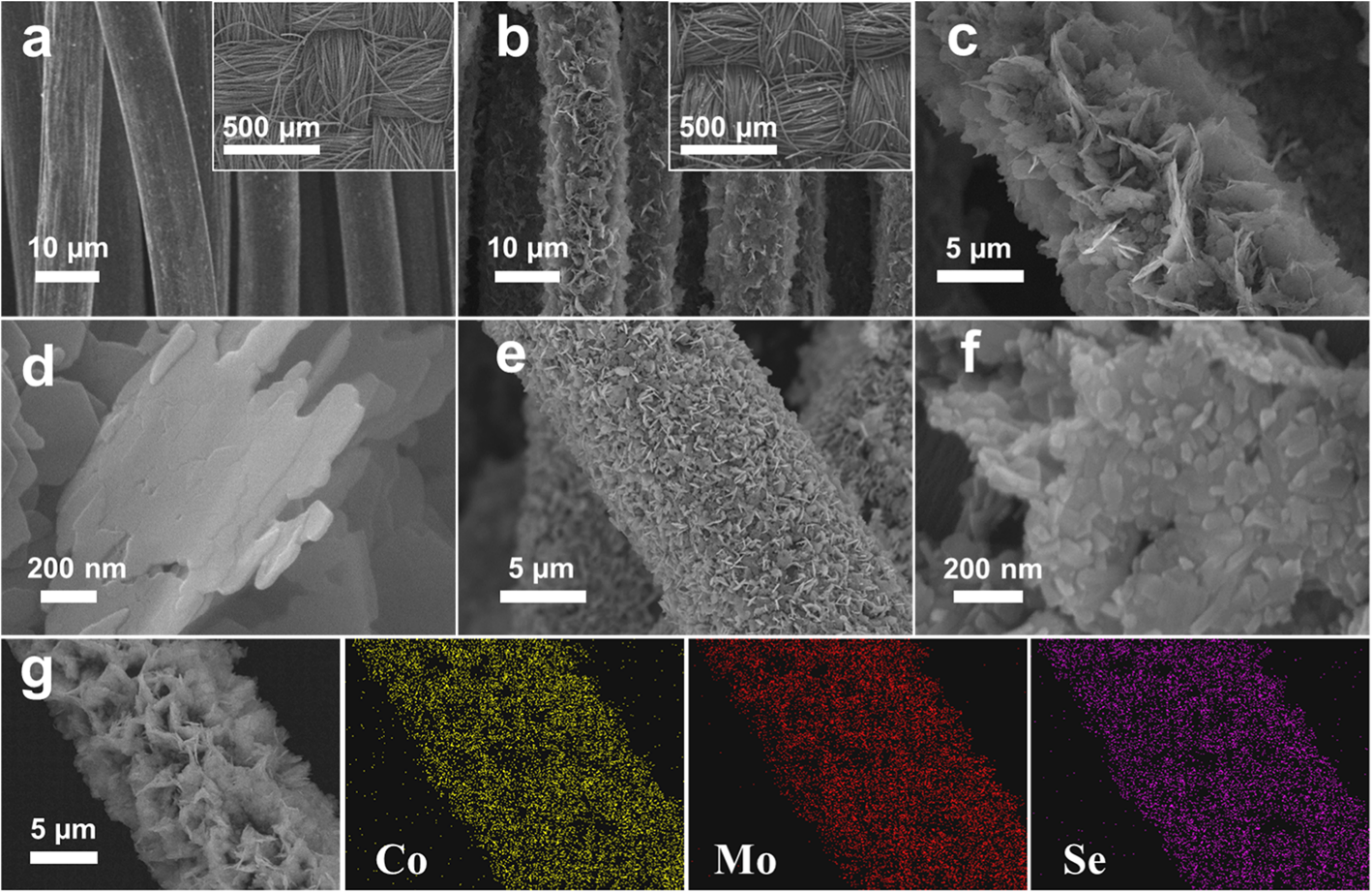
SEM images of **a** pure carbon cloth, **b**, **c**, **d** CoMoO_4_@C, and **e**, **f** CoMoSe_4_@C in different magnifications. **g** EDS elemental mappings of CoMoSe_4_@C

The synthesis of 3D CoMoSe_4_ nanosheet arrays converted directly from hydrothermally processed CoMoO_4_ by the plasma-assisted selenization process is schematically shown in Scheme 1. Basically, as a proof of concept, CoMoO_4_ nanosheets were grown on network fibers of a carbon cloth through hydrothermal process as displayed in Scheme 1a_1_, followed by the plasma-assisted selenization process as displayed in Scheme 1a_2_, CoMoO_4_@C directly converted into CoMoSe_4_ nanosheets. It can be demonstrated that the O atoms were nearly replaced by Se atoms after the plasma-assisted selenization process (Additional file 1: Figure S1). Detailed steps of CoMoSe_4_ nanosheets converted directly by the plasma-assisted selenization process were mentioned in the experimental part. Figure 1 a shows a SEM image of fibers taken from a carbon cloth where the inset shows a low-magnification SEM. After a hydrothermal process, CoMoO_4_ nanosheet arrays with a well-established texture structure were successfully grown on the fibers of the carbon cloth denoted as CoMoO_4_@C as shown in Fig. 1b. Figure 1 c shows a magnified SEM image taken from Fig. 1b where the nanosheet arrays with uniform in the diameter of approximately ~ 13 μm, consisting of high-density 3D nanosheets (Fig. 1d) with the networked morphology, can be clearly observed. After the plasma-assisted selenization under a power of 250 W at 450 °C for 1 h, nanosheet structures still remain as shown in Fig. 1e. However, there are slight changes in the morphology of the individual nanosheet, with which nanograins can be found on the surface instead of the smooth surface after the plasma-assisted selenization process as shown in Fig. 1f. The EDS elemental mapping images of Co, Mo, and Se on a randomly selected composite fiber as demonstrated in Fig. 1g soundly prove the successful production of CoMoSe_4_ on the carbon cloth with the uniform distribution around the individual fiber. Without the plasma-assisted treatment, the CoMoO_4_ cannot be completely converted into CoMoSe_4_ under the identical condition (250 W and 450 °C) as shown in Additional file 1: Figure S2a. These characteristic resonance modes of CoMoO_4_ still maintain after the selenization process without the plasma-assisted treatment (blue curve in Additional file 1: Figure S2a) while the black curve in Additional file 1: Figure S2a represents characteristic resonance modes of CoMoO_4_. Clearly, it can be expected that the Se radicals ionized from Se atoms by plasma-assisted treatment can speed up the chemical reaction between Co, Mo, and Se to form CoMoSe_4_ at the lower selenization temperature.

**Fig. 2:**
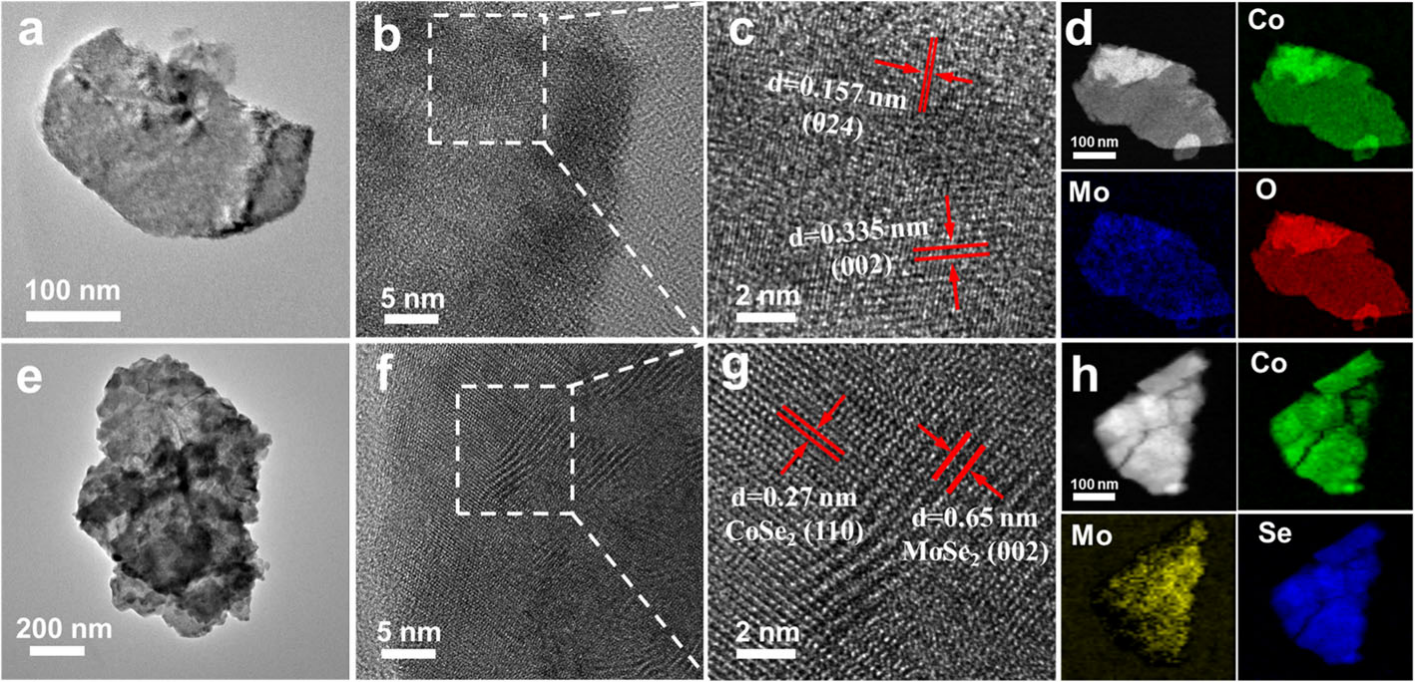
**a** A TEM image of CoMoO_4_ nanosheet. **b**, **c** HRTEM images of the CoMoO_4_ nanosheet in different magnifications. **d** The corresponding EELS elemental mappings of CoMoO_4_ nanosheet. **e** A TEM image of CoMoSe_4_ nanosheet. **f**, **g** HRTEM image of the CoMoSe_4_ nanosheet in different magnifications. **h** The corresponding EELS elemental mappings of CoMoSe_4_ nanosheet

Furthermore, TEM results also demonstrate the nanosheet morphology in the as-prepared CoMoO_4_ as shown in Fig. 2a, which is consistent with SEM images. In addition, a polycrystalline feature can be found in a CoMoO_4_ nanosheet where small single crystals can be well recognized by high-resolution TEM image as shown in Fig. 1b, c. As displayed in Fig. 1c, spaced lattice fringes in the distance of around 0.157 nm and 0.335 nm can be measured, which can be indexed to the crystal planes of (024) and (002), confirming the phase of CoMoO_4_. To further confirm the phase difference between CoMoO_4_ and CoMoSe_4_, Raman results were measured as shown in Additional file 1: Figure S2b. Before the plasma-assisted selenization process, the characteristic resonance modes at 330, 817, and 930 cm^−1^ are measured to well confirm the formation of the CoMoO_4_ phase (black curve in Additional file 1: Figure S2b) [44, 45]. However, significant changes in the corresponding resonance modes can be found in the Raman spectra before and after the plasma-assisted selenization process on the CoMoO_4_@C, with which the resonance mode at 168 cm^−1^ originated from CoSe_2_ [46], and typical MoSe_2_ features with E^1^_2g_ and A_1g_ modes located at 233 and 280 cm^−1^ verified the production of CoMoSe_4_ (red curve in Additional file 1: Figure S2b) [47]. The CoMoO_4_ and CoMoSe_4_ phases can be also evidenced by XRD spectra as shown in Additional file 1: Figure S3 where monoclinic CoMoO_4_ (JCPDS No. 21-0868), orthorhombic CoSe_2_ (JCPDS No. 53-0449), and hexagonal MoSe_2_ nanocrystals (JCPDS No. 29-0914**)** were confirmed, respectively. Additionally, the uniform distribution of Co, Mo, and O elements throughout the nanosheet can be confirmed by EDS elemental mapping images as shown in Fig. 2d indicating the homogeneous synthesis of the CoMoO_4_ after the hydrothermal process. The as-selenized CoMoSe_4_@C preserved the nanosheet structure, presenting the polycrystallinity as shown in Fig. 2e, f and characterized by Raman and XRD measurements (Additional file 1: Figures S2b and S3). The high-resolution TEM image as shown in Fig. 2g exhibits well-recognized lattice fringes separated by ~ 0.27 and ~ 0.65 nm, corresponding to (110) and (002) crystal planes of CoSe_2_ and MoSe_2_, respectively, confirming the successfully plasma-assisted selenization process to form the CoMoSe_4_. Similarly, the homogeneous transformation can be claimed with the uniform distribution of Co, Mo, and Se elements within the CoMoSe_4_ nanosheets as shown in Fig. 2h.

**Fig. 3:**
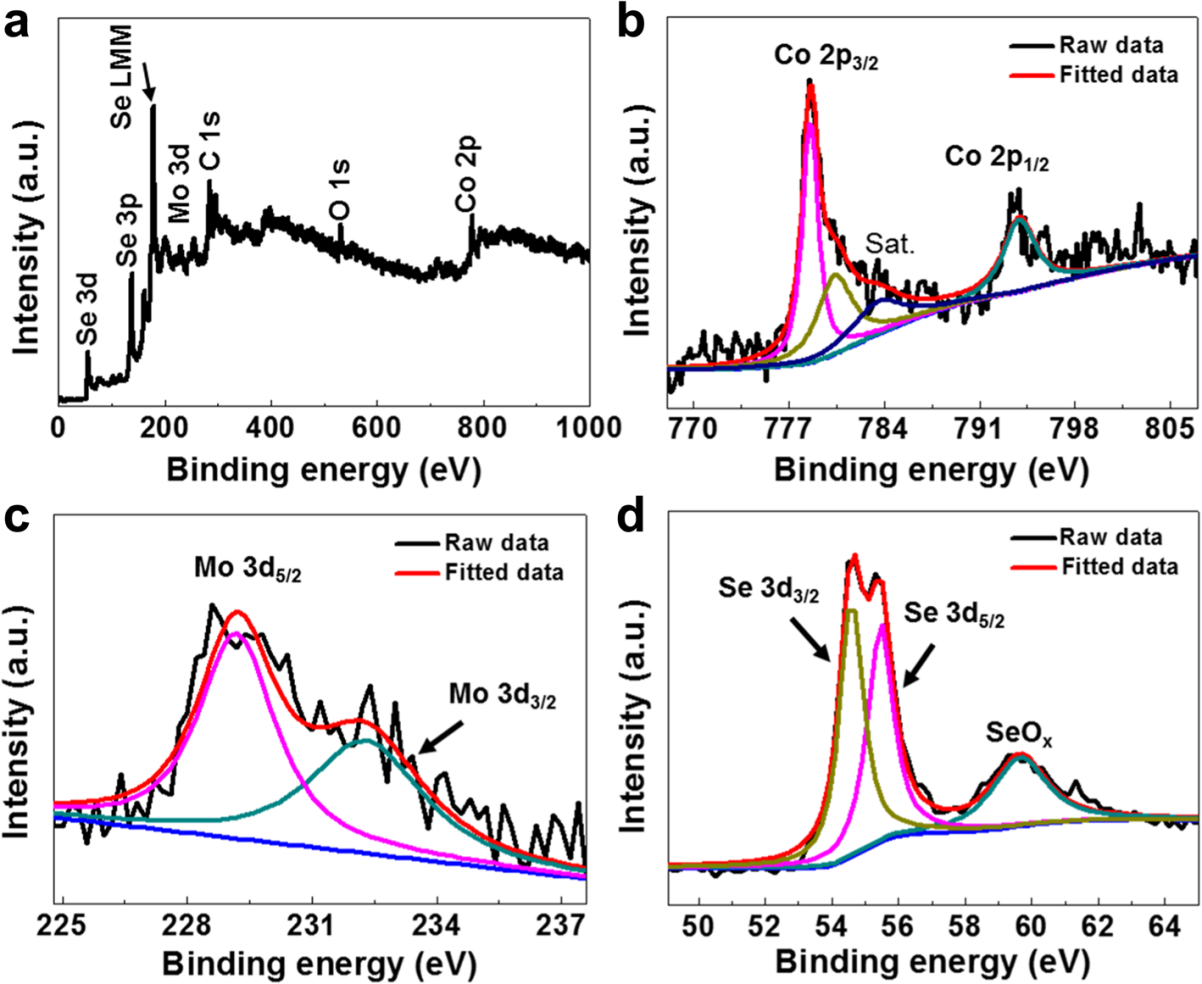
**a** The wide-scan XPS spectrum and narrow-scan spectra of **b** Co2p, **c** Mo3d, and **d** Se3d in the composite of CoMoSe_4_@C

In order to investigate the chemical composition of the selenized CoMoSe_4_, XPS measurements were carried out in the CoMoSe_4_@C composite, with which only Co, Mo, Se, C, and O elements can be identified within the instrumental limit as shown in Fig. 3a. Further narrow-scan spectra of Co 2p, Mo 3d, and Se 3d orbitals in both raw data and fitted curves were laid out in Fig. 3b–d. The 2p orbital-related peak of Co element splits into well-defined 2p_3/2_ and 2p_1/2_ peaks at 778.37 and 793.92 eV (Fig. 3b), suggesting that Co exists in the form of Co^2+^, and their satellite peaks marked as “Sat.” appeared at 780.37 and 783.52 eV, respectively [48, 49]. Two peaks at 232.25 and 229.53 eV (Fig. 3c) correspond to Mo 3d_3/2_ and Mo 3d_5/2_, indicating that Mo is in its Mo (IV) state [50, 51]. Additionally, peaks located at 54.59 and 55.46 eV in both raw data and fitted curves can be well-resolved corresponding to the Se 3d_5/2_ and Se 3d_3/2_ energies as shown in Fig. 3d [36, 52, 53]. Clearly, the peak observed at 59.64 eV is associated with SeO_x_, which was formed by the surface oxidation of CoMoSe_4_@C during sample handling [54]. The compositional analysis results show that the atomic ratio of Co:Mo:Se is about 1:0.88:3.84, indicating the stoichiometric of CoMoSe_4_.

**Fig. 4:**
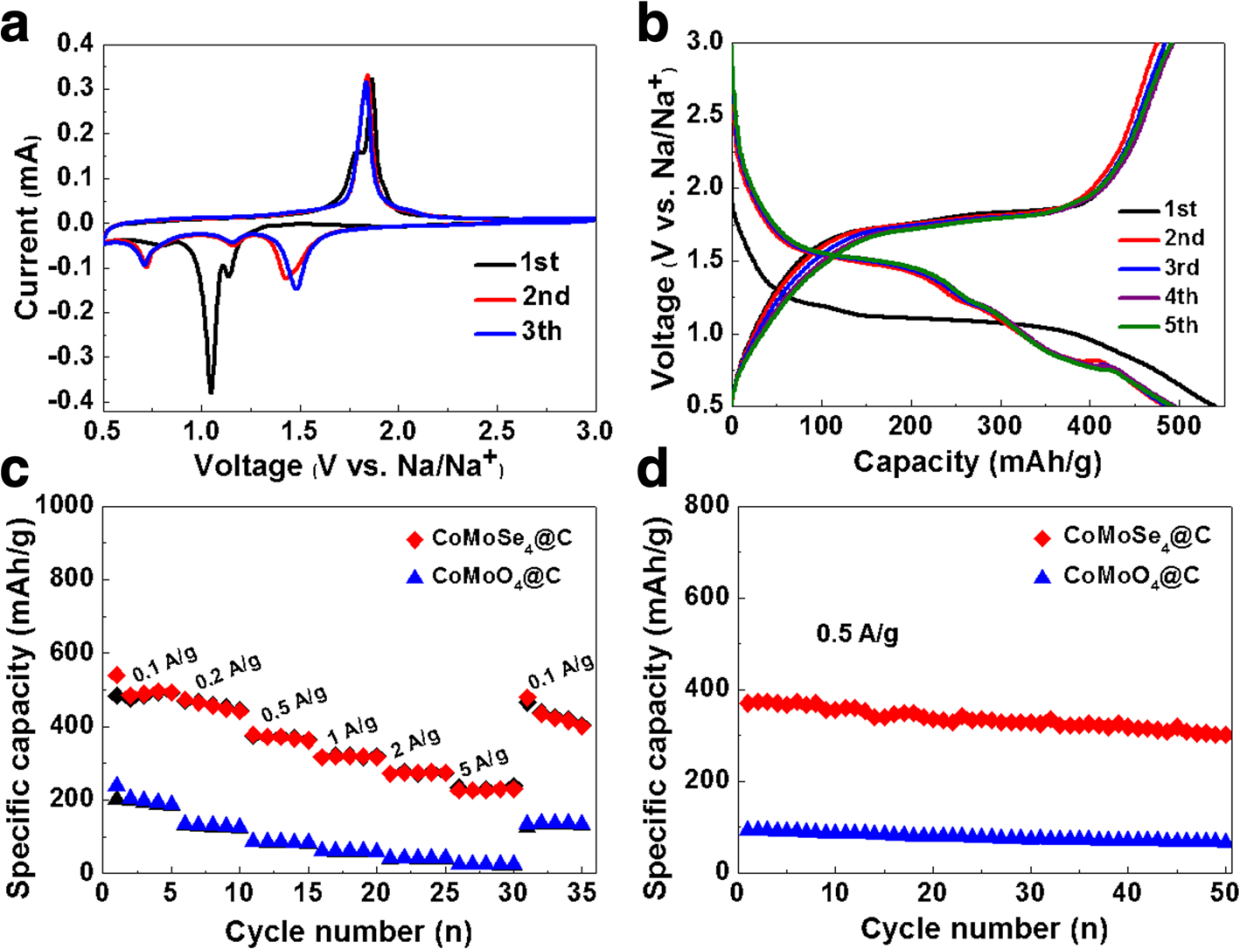
**a** CV curves of CoMoSe_4_@C at 0.1 mV s^−1^ under 0.5–3 V versus Na/Na^+^. **b** Discharge/charge curves of the CoMoSe_4_@C electrode within the first five cycles at 0.1 A g^−1^. **c** Capabilities of the CoMoSe_4_@C and CoMoO_4_@C electrodes at different charging rates under 0.5 to 3 V with its corresponding Coulombic efficiency. **d** Cycling performances of the CoMoSe_4_@C and CoMoO_4_@C electrodes for 50 cycles at 0.5 A g^−1^

The sodium storage performance of the CoMoSe_4_@C anode was evaluated using coin-type half cells with the unselenized CoMoO_4_@C electrode for the comparison. As can be seen in the cyclic voltammogram (CV) curves of the CoMoSe_4_@C electrode as shown in Fig. 4a, two peaks at ~ 1.14 and 1.05 V during the first cathodic sweep can be resolved corresponding to the insertion process by Na^+^ ion while the two oxidation peaks at around 1.79 V and 1.86 V are related to the extraction process of the Na^+^ ion. Starting from the second cycles, the CV curves in this composite as the anode material exhibit overlaps with the subsequent cycle, indicating the good electrode stability. The corresponding galvanostatic charge/discharge curves as displayed in Fig. 4b are consistent with the CV results and demonstrate the stable Na^+^ insertion/extraction behaviors within the first five cycles except for some irreversible reactions. It should be mentioned that the fiber structure of the carbon cloth almost contributed nothing in the capacity evidenced by the cycling measurements as shown in Additional file 1: Figure S4. For the comparison, CV and charge/discharge curves of the CoMoO_4_@C electrode at the same measured conditions are displayed in Additional file 1: Figure S5. Note that the poor electrochemical activity in the CoMoO_4_@C composite as the anode for SIBs can be confirmed. Undoubtedly, the plasma-assisted selenization of CoMoO_4_@C is quite constructive to produce more suitable electrode materials for sodium storage.

Additional file 1: Figure S6 shows the raw experimental and fitted Nyquist plots for CoMoSe_4_@C and CoMoO_4_@C as well as the corresponding equivalent circuit (inset of Additional file 1: Figure S6). Clearly, the fitting results confirm that the charge transfer resistance (Rct) of CoMoSe_4_@C and CoMoO_4_@C is 19 and 157 Ω, respectively. EIS results reveal the electrochemical mechanisms of improved sodium storage capability in the CoMoSe_4_@C composite electrode, in which the better electrical conductivity can be characterized in the plasma-assisted selenized composite to facilitate the faster Na^+^ insertion/extraction even at high current densities than those in the CoMoO_4_@C composite. Moreover, the superior rate performance in the CoMoSe_4_@C electrode was accomplished comparing with the CoMoO_4_@C as demonstrated in Fig. 4c with the current densities stepwisely increasing from 0.1 to 5 A g^−1^. Specifically, a high reversible capacity of 475 mA h g^−1^ at 0.1 A g^−1^ was shown by the CoMoSe_4_@C electrode with the contrast to only 198 mA h g^−1^ in the CoMoO_4_@C anode. As current densities are stepwisely increased from 0.1 to 0.2, 0.5, 1, 2, and 5 A g^−1^, reversible capacities in the CoMoSe_4_@C electrode dropped from 475 to 458, 371, 320, 277, and 230 mA h g^−1^, indicating the good rate capability. As a comparison, the discharge capacities in the CoMoO_4_@C anode experienced a reduction from 198 to 140, 93, 65, 45, and 26 mA h g^−1^, respectively. Furthermore, the similar phenomena can be found in the cycling testing results of the CoMoSe_4_@C and CoMoO_4_@C electrodes as presented in Fig. 4d. The CoMoSe_4_@C exhibited a better cycling stability with a high capacity of 301 mA h g^−1^ at 0.5 A g^−1^ maintained even after 50 cycles compared to 46 mA h g^−1^ in the CoMoO_4_@C electrode. Compared with the anode materials previously reported (Table 1), CoMoSe_4_@C composite electrode exhibits considerable reversible capacity and rate performance, thus CoMoSe_4_@C composite can be used as a potential electrode material for SIBs.

**Table 1:** Electrochemical properties of various anode materials applied as sodium-ion batteries reported in the previous literature

Materials description	Voltage range (V vs Na^+^/Na)	Specific capacity (mAh g^−1^)/current density	Cycling data (mAh g^−1^)/cycles/current density	Reference
MoSe_2_/CNT	0.001–2.5 V	382/0.2 A g^−1^	296/250th/1 A g^−1^	[31]
CoSe_2_@N-PGC/CNTs	0.001–3 V	482/0.2 A g^−1^	424/100th/0.2 A g^−1^	[32]
FeSe_2_	0.5–2.9 V	447/0.1 A g^−1^	372/2000th/1 A g^−1^	[24]
MoO_2_@MoSe_2_	0.01–3 V	1136/0.1 A g^−1^	520.4/400th/2 A g^−1^	[36]
CoSe_2_	0.001–3 V	521/0.1 A g^−1^	467/40th/0.5 A g^−1^	[25]
CoSe_2_/(NiCo)Se_2_	0.001–3 V	554/0.2 A g^−1^	497/80th/0.2 A g^−1^	[29]
Ni_0.85_Se/C	0.01–3 V	397/0.2 A g^−1^	390/100th/0.2 A g^−1^	[26]
WSe_2_/C	0.01–3 V	294/0.1 A g^−1^	270/50th/0.2 A g^−1^	[12]
MoSe_2_/Gr	0.01–3 V	432/0.1 A g^−1^	380/50th/0.4 A g^−1^	[27]
SnSe/RGO	0.01–2 V	500/0.1 A g^−1^	385/50th/0.5 A g^−1^	[13]
Sb_2_Se_3_@N-Gr	0.01–3 V	705/0.1 A g^−1^	548.6/50th/0.2 A g^−1^	[14]
VSe_2_/Gr	0.01–3 V	559/0.2 A g^−1^	632/60th/0.1 A g^−1^	[15]
CoMoSe_4_@C	0.5–3 V	475/0.1 A g^−1^	301/50th/0.5 A g^−1^	This work

## Conclusions

A facile approach to prepare a binary transition metallic selenide to serve as the anode material in SIBs was demonstrated via the plasma-assisted selenization process of a binary transition metallic oxide. In this work, three-dimensional (3D) CoMoSe_4_ nanosheets on network fibers of a carbon cloth denoted as CoMoSe_4_@C directly converted from CoMoO_4_ nanosheets prepared by hydrothermal process on network fibers of a carbon cloth through the plasma-assisted selenization as the anode for SIBs were demonstrated for the first time. A large sodium-ion storge of 475 mA h g^−1^ at 0.1 A g^−1^ can be generated from the plasma-assisted selenized composite electrode with the capacity retention of over 80% maintained even after 50 cycles, while the discharge capacity of 230 mA h g^−1^ still can be obtained even at 5 A g^−1^. Excellent Na-ion storage capabilities benefit from its well-developed nanostructure and good electrical conductivity. The work highlights the promising application of binary transition metallic selenides as electrode materials in SIBs and the simple synthesis method which might be employed in the production of other bimetallic selenides for a variety of applications, such as powering sustainable vehicles and portable energy storage devices.

## Additional File

Additional file 1: **Figure S1.** SEM-energy dispersive spectra (EDS) of (a) CoMoO_4_@C and (b) CoMoSe_4_@C. **Figure S2.** Raman spectra of (a) CoMoO_4_ before and after the plasma-assisted selenization process without plasma treatment and (b) CoMoO_4_ before and after the plasma-assisted selenization process with plasma treatment. **Figure S3.** XRD spectra of (a) CoMoO_4_ and (b) CoMoSe_4_ nanosheet arrays. **Figure S4.** Cycling performance of pure carbon cloth. **Figure S5.** (a) Cyclic voltammograms and (b) discharge/charge profiles of the CoMoO_4_@C. **Figure S6.** EIS of CoMoSe_4_@C and CoMoO_4_@C. (DOCX 357 kb)

## Data Availability

All data generated or analyzed during this study are included in this published article and its supplementary information files.

## Abbreviations

3D: Three-dimensional
CoMoO_4_@C: 3D CoMoO_4_ nanosheets/carbon cloth
CoMoSe_4_@C: 3D CoMoSe_4_ nanosheets/carbon cloth
CV: Cyclic voltammetry
EELS: Electron energy loss spectroscopy
EIS: Electrochemical impedance spectroscopy
LIBs: Lithium-ion batteries
MXs: Metal sulfides/selenides
SEM: Scanning electron microscopy
SIBs: Sodium-ion batteries
TEM: Transmission electron microscopy
XPS: X-ray photoelectron spectroscopy
XRD: X-ray diffraction
