# Supplementary material for: 3D CoMoSe4 Nanosheet Arrays Converted Directly from Hydrothermally Processed CoMoO4 Nanosheet Arrays by Plasma-Assisted Selenization Process Toward Excellent Anode Material in Sodium-Ion Battery
Source: Nanoscale Res Lett. 2019 Jun 25;14:213. doi: 10.1186/s11671-019-3035-6 (PMC6593019; doi:10.1186/s11671-019-3035-6)
Supplement: Supplementary file 1 — Figure S1. SEM-energy dispersive spectra (EDS) of (a) CoMoO4@C and (b) CoMoSe4@C. Figure S2. Raman spectra of (a) CoMoO4 before and after the plasma-assisted selenization process without plasma treatment and (b) CoMoO4 before and after the plasma-assisted selenization process with plasma treatment. Figure S3. XRD spectra of (a) CoMoO4 and (b) CoMoSe4 nanosheet arrays. Figure S4. Cycling performance of pure carbon cloth. Figure S5. (a) Cyclic voltammograms and (b) discharge/charge profiles of the CoMoO4@C. Figure S6. EIS of CoMoSe4@C and CoMoO4@C. (DOCX 357 kb) [file 11671_2019_3035_MOESM1_ESM.docx]

**Supporting Information**

**3D CoMoSe_4_ Nanosheet Arrays Converted Directly From Hydrothermally Processed-CoMoO_4_ Nanosheet Arrays by Plasma-Assisted Selenization Process Toward Excellent Anode Material in Sodium-ion Battery**

Shan Zhang,^ab^ Yuanfei Ai,^b^ Shu-Chi Wu,^b^ Hsiang-Ju Liao,^b^ Teng-Yu Su,^b^ Jyun-Hong Chen,^b^ Chuan-Hsun Wang,^b^ Ling Lee,^b^ Yu-Ze Chen,^b^ Binbin Xu,^d^ Shin-Yi Tang,^b^ Ding Chou Wu,^b^ Shao-Shin Lee,^b^ Jun Yin, ^a^Jing Li*^a^, Junyong Kang ^a^ and Yu-Lun Chueh*^bce^

^a^Collaborative Innovation Center for Optoelectronic Semiconductors and Efficient Devices, Pen-Tung Sah Institute of Micro-Nano Science and Technology/Department of Physics, Xiamen University, Xiamen, Fujian 361005, China

^b^Department of Materials Science and Engineering, National Tsing Hua University, Hsinchu 30013, Taiwan, Republic of China

^c^Frontier Research Center on Fundamental and Applied Sciences of Matters, National Tsing Hua University, Hsinchu 30013, Taiwan, Republic of China

^d^College of Chemistry and Chemical Engineering, Xiamen University, Xiamen, Fujian 361005, China

^e^Department of Physics, National Sun Yat-Sen University, Kaohsiung, 80424, Taiwan, ROC

*Corresponding emails: lijing@xmu.edu.cn and ylchueh@mx.nthu.edu.tw

**
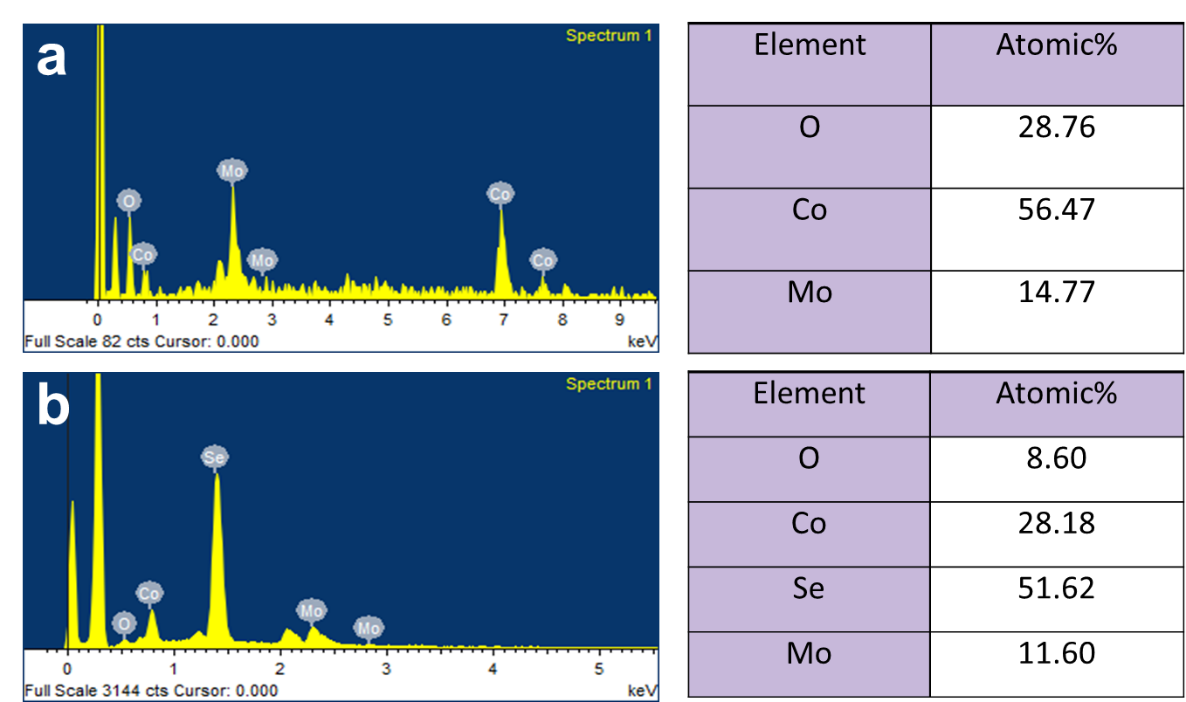
**

Figure S1 SEM-energy dispersive spectra (EDS) of (a) CoMoO_4_@C and (b) CoMoSe_4_@C.

**
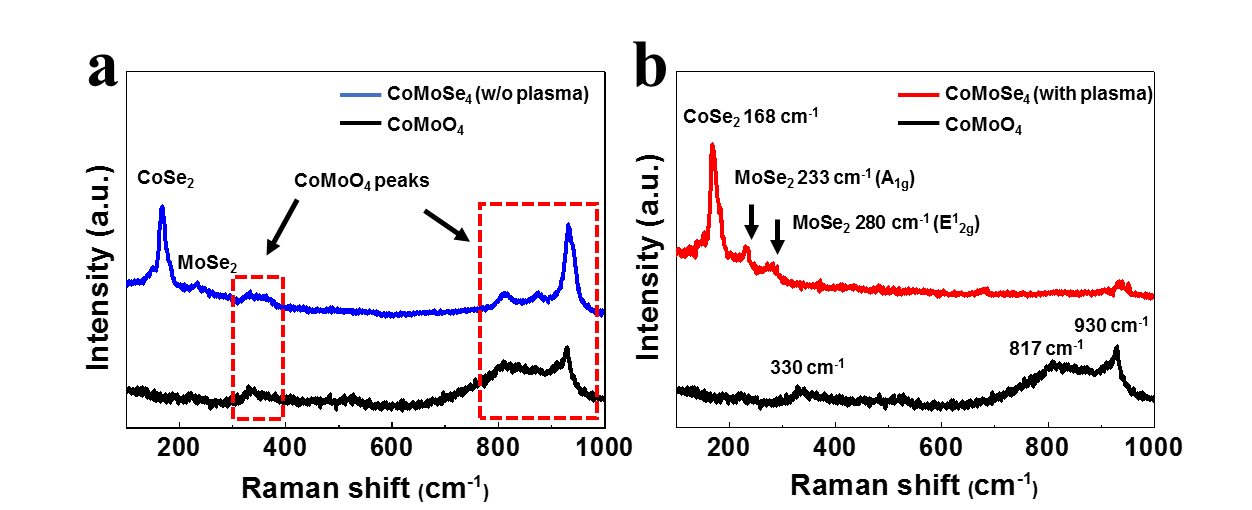
**

Figure S2 Raman spectra of (a) CoMoO_4_ before and after the plasma-assisted selenization process without plasma treatment and (b) CoMoO_4_ before and after the plasma-assisted selenization process with plasma treatment.


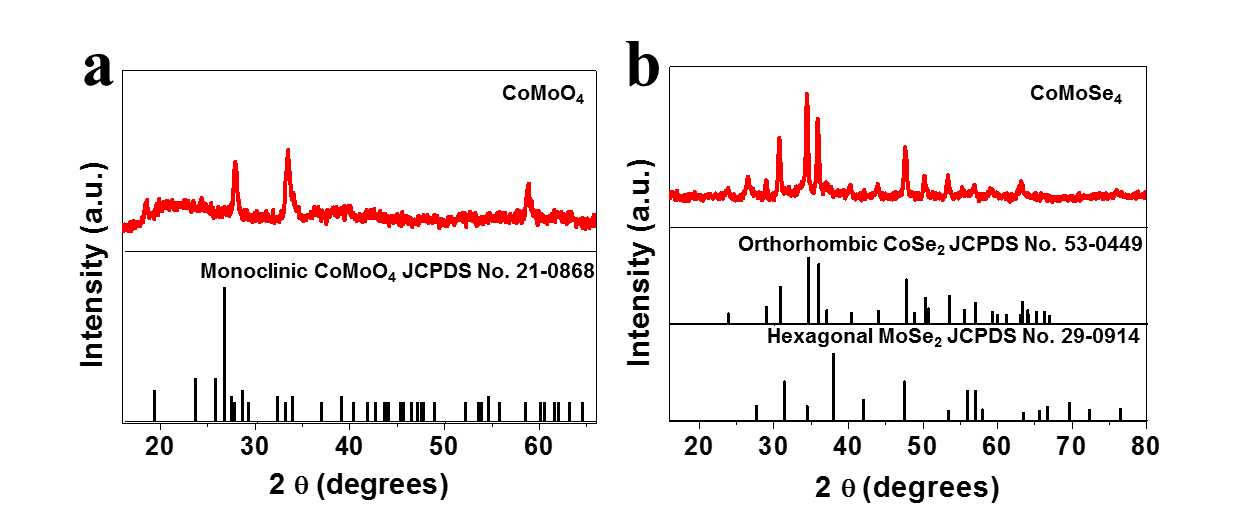


Figure S3 XRD spectra of (a) CoMoO_4_ and (b) CoMoSe_4_ nanosheet arrays.

Figure S4. Cycling performance of pure carbon cloth.


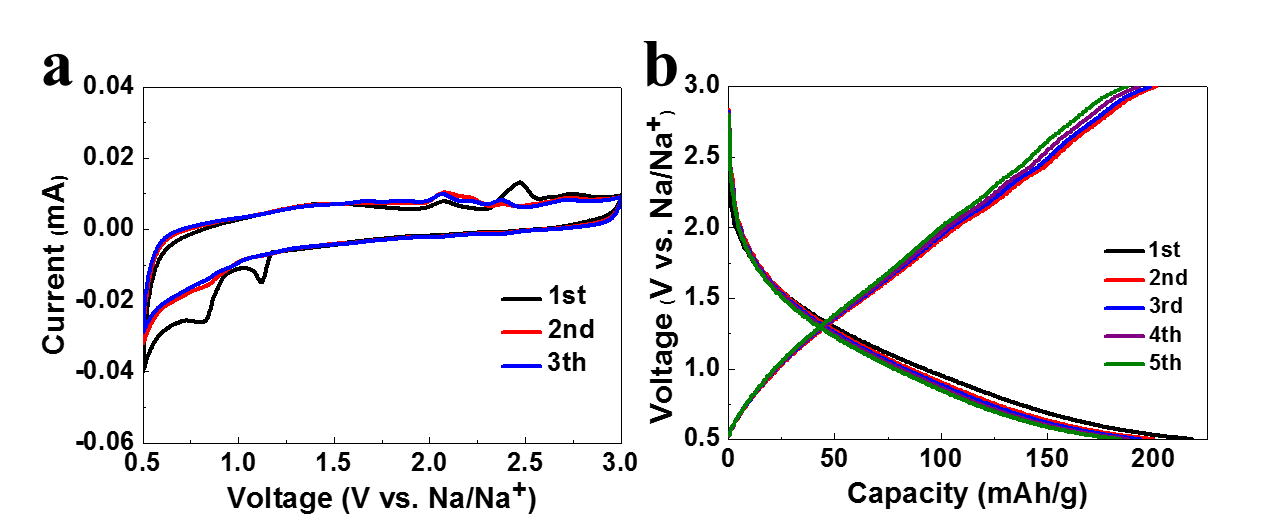


Figure S5. (a) Cyclic voltammograms and (b) Discharge/charge proﬁles of CoMoO_4_@C.


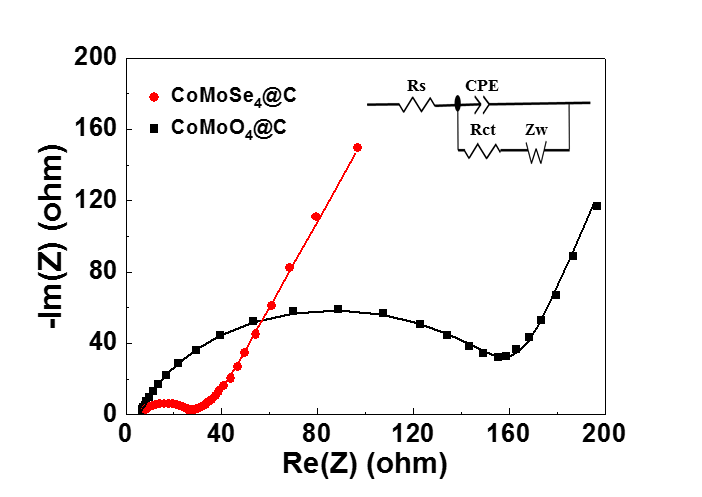


Figure S6. EIS results of CoMoSe_4_@C and CoMoO_4_@C.
